# Does implant drill design influence the accuracy of dental implant placement using static computer-assisted implant surgery? An in vitro study

**DOI:** 10.1186/s12903-023-03297-0

**Published:** 2023-08-18

**Authors:** Anna Takács, Gyula Marada, Kinga Turzó, Ákos Nagy, Orsolya Németh, Eitan Mijiritsky, Márton Kivovics, Attila Mühl

**Affiliations:** 1 Department of Community Dentistry, Semmelweis University, Szentkirályi utca 40, Budapest, 1088 Hungary; 2 Dental School, Medical Faculty, University of Pécs, Tüzér utca 1, Pécs, 7623 Hungary; 3 Department of Head and Neck Surgery and Maxillofacial Surgery, Tel-Aviv Sourasky Medical Center, School of Medicine, Tel-Aviv University, Tel Aviv, 64239 Israel; 4 Goldschleger School of Dental Medicine, Faculty of Medicine, Tel-Aviv University, Tel Aviv, 39040 Israel

**Keywords:** Computer-assisted implant surgery (CAIS), Dental implant, Surgical guide, Static navigation, Implant drill, Step drill, Straight drill, In vitro study

## Abstract

**Background:**

The purpose of this in vitro study was to compare the accuracy of implant placement in model surgeries according to the design of the drills (straight drills or step drills) used to finalize the implant bed during pilot-guided static computer-assisted implant surgery (sCAIS).

**Methods:**

Model surgeries were carried out on resin models randomly assigned to three study groups. Virtual planning software (coDiagnostiX 10.6, Dental Wings, Montreal, Canada) was used to plan the implant positions. In Groups 1 and 2, pilot-guided sCAIS was performed. Straight drills were used in Group 1, and step drills were used in Group 2 to finalize the implant beds. In Group 3, fully guided sCAIS was performed using a universal fully guided kit (RealGUIDE Full Surgical Kit 3DIEMME, RealGUIDE, Cantù, Como, Italy). A total of 90 dental implants (Callus Pro, Callus Implant Solutions GmbH, Nuremberg, Germany) were placed (six implants per model, five models per study group). The primary outcome variables (angular deviation, coronal global deviation, and apical global deviation) were calculated for all implants based on the comparison of the preoperative surgical plan with the postoperative scans.

**Results:**

Group 2 (coronal global deviation, 0.78 ± 0.29 mm; apical global deviation, 1.02 ± 0.56 mm) showed significantly lower values of both global deviation variables than Group 1 (coronal global deviation, 0.95 ± 0.20 mm; apical global deviation, 1.42 ± 0.49 mm). However, there was no significant difference in angular deviation between Groups 1 and 2 (7.56 ± 2.92° and 6.44 ± 2.84°). Group 3 produced significantly lower values of all three primary outcome variables (angular deviation, 2.36 ± 0.90°; coronal global deviation, 0.59 ± 0.28 mm; apical global deviation, 0.90 ± 0.29 mm) than Group 1 and significantly lower angular deviation and coronal global deviation values than Group 2.

**Conclusions:**

The design of the drills used to finalize implant osteotomies during pilot-guided sCAIS influences dental implant placement accuracy. Using step drills instead of straight drills for final osteotomies decreases deviation from the surgical plan. The fully guided approach performed better than the pilot-guided sCAIS.

**Supplementary Information:**

The online version contains supplementary material available at 10.1186/s12903-023-03297-0.

## Background

Backwards planning in oral implantology enables the clinician to conceive the number, position, and angulation of dental implants based on the prosthetic plan [1]. Contemporary digital solutions allow for registration of cone beam computed tomography (CBCT) data, digital impressions, and face scans, thereby assisting in planning dental implant prosthetics [2–6]. Computer-assisted implant surgery (CAIS) enables the transfer of the planned implant positions to the surgical site [7, 8].

Guided implant placement allows the surgeon to perform an intervention with decreased invasiveness compared to the free-hand method, which reduces postoperative morbidity [9]. In static CAIS (sCAIS), a surgical template is manufactured through virtual planning to guide the drills during implant bed preparation [10]. In a fully guided procedure, every step of the implant bed preparation, as well as the implant placement, is guided by the surgical template. During a half-guided protocol, the final steps of implant bed preparation and implant placement are carried out without the guidance of the surgical template. The pilot-guided protocol is a subtype of half-guided sCAIS, where only the first osteotomy is guided; the template is then removed, the implant bed is finalized, and the implant is placed in a free-handed manner [11–13]. Accurate positioning of the platform of the implant helps the surgeon reduce complications in the adjacent teeth and preserve sufficient bone on the vestibular and palatal aspect of the implant, which facilitates the long-term stability of soft and hard tissues surrounding the dental implant. Decreasing deviations at the apical part of the dental implant enable the clinician to avoid the nasal cavity, maxillary sinuses, nerves, and major blood vessels. Optimal angulation of the implant facilitates ideal biomechanics and acceptable aesthetics of the prosthesis [14–16].

According to the results of the meta-analysis of Gargallo-Albiol et al. based on ten randomized clinical studies, a 0.51 mm (95% confidence interval (CI) [0.29, 0.73]) coronal global deviation, 0.75 mm (95% CI [-1.19, -0.31]) apical global deviation, and 3.63° (95% CI [-4.10, -3.16]) benefit in accuracy is observed in the literature, favouring fully guided surgery over half-guided surgery [17]. However, as the surgical template remains in place for the whole intervention, cooling of the drills is inhibited during fully guided sCAIS [18, 19]. The heating of the surgical template and its resulting dimensional changes may affect implant placement accuracy [20]. Another factor influencing the accuracy of sCAIS is the material of the surgical template and its biomechanical properties [21, 22]. Pilot-guided sCAIS enables abundant cooling throughout the surgery and requires less elaborate instrumentation, as the surgeon finalizes the implant bed using drills and instructions provided by the manufacturer of the dental implant to be placed [7, 23]. Several dental implant manufacturers provide straight drills in their surgical trays for implant bed preparation. However, some manufacturers equip the trays with step drills. The diameter of the apical portion of these step drills corresponds with the coronal diameter of the previous drill in the drilling sequence, aiming to prevent deviation from the angulation already determined by earlier osteotomies [24].

There is a lack of evidence in the literature on whether using step drills or straight drills for the finalization of the implant bed during pilot-guided sCAIS influences the accuracy of dental implant placement.

This in vitro study aimed to compare the accuracy of implant placement in model surgeries according to the design of the drills (straight drills or step drills) used to finalize the implant bed during pilot-guided sCAIS. Our null hypothesis was that there is no significant difference in the accuracy of implant placement achieved whether straight drills or step drills are used to finalize implant beds following the guided pilot osteotomy. In addition, we hypothesized that fully guided sCAIS allows for more accurate implant positioning compared to the pilot-guided intervention regardless of the design of the drills used to finalize implant osteotomies.

## Materials and methods

### Study design

An in vitro study was designed to test our hypotheses. The sample size was determined based on the results of Stünkel et al. [25] and Kivovics et al. [26] using G*Power 3.1 software (v.3.1.9.3, 2017, Institut für Experimentelle Psychologie, Heinrich-Heine-Universität, Düsseldorf, Germany). According to the results of these studies, an angular deviation of 4.6 ± 1.50° may be expected from a pilot-guided implant placement protocol where implant beds were finalized using straight drills, whereas an angular deviation of 3.21 ± 1.52° may be expected from pilot-guided sCAIS where step drills were used to finalize osteotomies. If α (false-positive rate) was set at 0.05, to reach a power of 95% with a 1:1 distribution ratio between study groups, the minimal sample size was determined to be at least 27 dental implants placed per study group.

In Group 1, model surgeries were carried out using pilot-guided sCAIS followed by step drills. In Group 2, implant placement was performed with a pilot-guided protocol followed by straight drills. In Group 3, fully guided sCAIS was performed to place implants. The brand, diameter, and length of the implants used were the same in all three study groups and at all sites (4.2 mm diameter, 10 mm length, article number: CP 1042 − 100, Callus Pro, Callus Implant Solutions GmbH, Nuremberg, Germany).

To minimize participant bias, different examiners were tasked with performing individual assignments. The clinician assigned to surgical planning (A.M.) did not take part in the surgical interventions and data acquisition. The clinician performing the model surgery (M.K.) was not involved in the evaluation of the accuracy of implant placement (A.T.). The examiner carrying out the measurements of the outcome variables (A.T.) was blinded to the surgical modality used during the model surgeries.

### Preoperative CBCT, digital impression, and implant planning

Resin models for hands-on training and education mimicking D3-type bone according to the Misch classification (U-009 A, BoneModels S.L.U., Castellon, Spain) were randomly assigned to three study groups.

CBCT scans (Planmeca Promax 3D, Planmeca, Helsinki, Finland) were carried out with an 8 × 8 cm field of view at a voxel size of 200 μm, exposure time of 12.45 s, tube voltage of 62 kV, and tube current of 5 mA prior to the model surgeries. The models were digitized to Standard Tessellation Language (STL) files using a desktop scanner (Medit T710, Medit, Seoul, Republic of Korea).

The remaining dentition on the models consisted of the upper incisors. Prosthetic planning was performed using DWOS 16.0.1 software (Dental Wings, Montreal, Canada, USA) to replace the missing teeth (World Dental Federation, FDI 13, 14, 15, 16, 23, 24, 25, and 26) with four-unit, screw-retained fixed partial dentures on both sides with dental implants at the sites of the canines, first premolar, and first molar. Virtual planning of the positions of the dental implants (4.2 mm diameter, 10 mm length, CP 1042 − 100, Callus Pro, Callus Implant Solutions GmbH, Nuremberg, Germany) and surgical templates was carried out using coDiagnostiX 10.6 software (Dental Wings, Montreal, Canada). STL files of the models were registered with the Digital Imaging and Communications in Medicine (DICOM) data of the CBCT reconstructions. Surgical templates for the pilot-guided interventions of Groups 1 and 2 were designed with sleeves of a 2.0 mm inner diameter, 4.0 mm outer diameter, and height of 4.0 mm (article number: GSLEEVE, Callus Implant Solutions GmbH, Nuremberg, Germany). Sleeves were positioned 19.2 mm from the planned position of the implant apex at each site, resulting in a free drilling distance of 15.2 mm to correspond with the free drilling distance of the surgical templates used in Group 3. Three sleeves for the guide fixation pins (article number: 3DM00671, 3DIEMME, RealGUIDE, Cantù, Como, Italy) were planned to stabilize the surgical guides.

Surgical templates for the fully guided procedures of Group 3 were planned with sleeves (article number: 3DM00604CAD1.10, 3DIEMME, RealGUIDE, Cantù, Como, Italy) specific to the universal fully guided kit and three sleeves for the guide fixation pins similar to the ones used in Groups 1 and 2. The sleeves available for this fully guided system (article number: 3DM00604CAD1.10, 3DIEMME, RealGUIDE, Cantù, Como, Italy) have an inner diameter of 5.0 mm, an outer diameter of 6.0 mm and a height of 4.0 mm. The shank of the drills used in this system is modified to fit inside the metal sleeve in the surgical template. All six sleeves were planned to be placed 19.2 mm from the planned position of the implant apex, resulting in a free drilling distance of 15.2 mm.

Rapid prototyping (3D printing) of the surgical templates was carried out using Form 2 hardware (Formlabs, Somerville, Massachusetts, USA) and Clear Resin material (Formlabs, Somerville, Massachusetts, USA) in all three study groups.

### Model surgery

The interventions were performed by a surgeon (M.K.) who was well experienced in the clinical use of static CAIS with both pilot-guided and fully guided protocols. Model surgeries were carried out in a randomized order over the course of five days with three model surgeries (18 implants placed) each day to avoid inaccuracies due to fatigue.

Osteotomies were carried out at a drill rotation speed of 800 rpm with external cooling. Dental implants 4.2 mm in diameter and 10 mm in length (article number: CP 1042 − 100, Callus Pro, Callus Implant Solutions GmbH, Nuremberg, Germany) were placed at sites FDI 13, 14, 16, 23, 24, and 26 in all three study groups.

In Group 1, the fit of the surgical guide was verified, and the templates were stabilized on the models using three guide fixation pins (article number: 3DM00609, 3DM00671, 3DIEMME, RealGUIDE, Cantù, Como, Italy). A pilot drill with a 2 mm diameter (article number: CP 2mmL, Callus Implant Solutions GmbH, Nuremberg, Germany) was used for the pilot osteotomy. The surgical template was removed, and straight implant drills of 2.3 mm, 2.8 mm, 3.4 mm, and 3.8 mm (article numbers: CP 2.3mmL, CP 2.8mmL, CP 3.4mmL, CP 3.8mmL, Callus Implant Solutions GmbH, Nuremberg, Germany) were used for implant bed preparation. Dental implant placement was performed manually with a ratchet.

In Group 2, after checking the fit of the surgical template, stabilization was carried out using three guide fixation pins. A pilot drill with a 2 mm diameter (article number: CP 2mmL, Callus Implant Solutions GmbH, Nuremberg, Germany) was used for the pilot osteotomy. Implant beds were finalized in a free-handed manner according to the manufacturer’s instructions. Step drills of 2.0/2.3 mm, 2.3/2.85 mm, 2.8/3.3 mm, and 3.25/3.7 mm (article numbers: CP CD1, CP CD2, CP CD3, CP CD4, Callus Implant Solutions GmbH, Nuremberg, Germany) were used for osteotomies. Dental implant placement was performed manually with a ratchet.

**Fig. 1:**
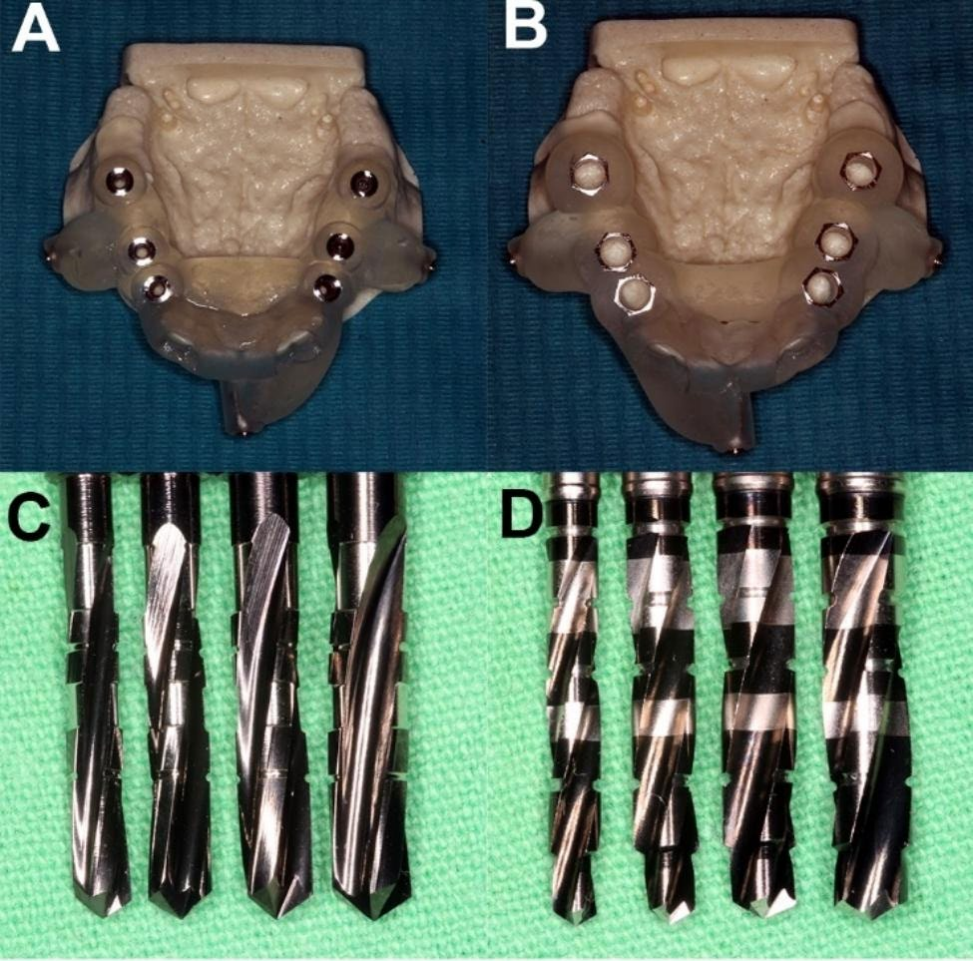
The experimental setup in the three study groups. In Group 1, model surgeries were carried out using pilot-guided sCAIS followed by step drills. In Group 2, implant placement was performed with a pilot-guided protocol followed by straight drills. In Group 3, fully guided sCAIS was performed to place implants. The design of one of the surgical templates used in Groups 1 and 2 (**A**) and Group 3 (**B**). Set of straight (**C**) and step (**D**) drills used to finalize implant beds in Groups 1 and 2

In Group 3, the fit of the template was checked, and fully guided implant bed preparation was carried out according to the instructions of the manufacturer of the fully guided kit (RealGUIDE Full Surgical Kit, RealGUIDE, Cantù, Como, Italy) using the following drill sequence: bone crest leveller (article number: 3DM00614), start drill, (article number: 3DM00621), drill D1.6 × 7 mm (article number: 3DM00613.16.70), drill D2.3 × 8.5 mm (article number: 3DM00613.23.85), drill D2.3 × 10 mm (article number: 3DM00613.23.10), drill D3.0 × 10 mm (article number: 3DM00613.30.10), drill D3.4 × 10 mm (article number: 3DM00613.34.10), and drill D3.8 × 10 mm (article number: 3DM00613.38.10). Implant placement was performed using a manual torque wrench (article number: 3DM00611) with the implant mount (Nobel Active RP connection, article number: 3DM00606NBLACTRPL 3DIEMME, RealGUIDE, Cantù, Como, Italy). Figure 1 presents the experimental setup in the three study groups.

### Postoperative imaging

Scan bodies (cp52.042, Callus Implant Solutions GmbH, Nuremberg, Germany) were connected to the implants, and postoperative scanning of the models was carried out using a desktop scanner (Medit T710, Medit, Seoul, Republic of Korea). Computer-aided design (CAD) files of the scan body and the dental implant were acquired from the implant manufacturer (Callus Implant Solutions GmbH, Nuremberg, Germany).

### Trueness evaluation

**Fig. 2:**
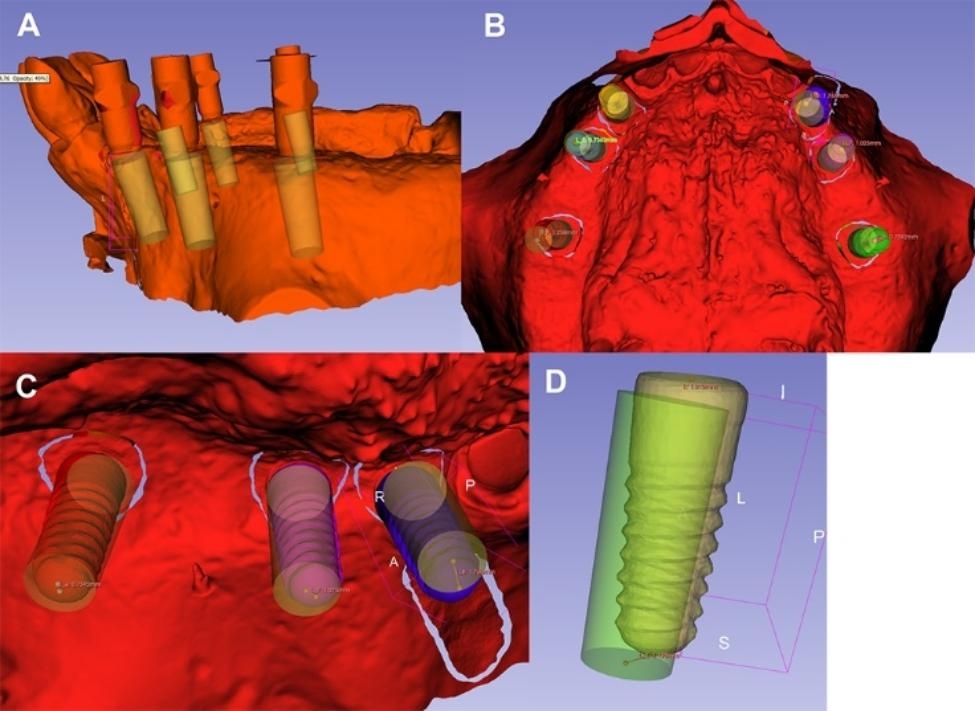
Outcome variables were measured in Slicer 5.2.2 (The Brigham and Women’s Hospital, Inc., Boston, USA). Cylinders represent the planned implant positions, whereas the dental implant surfaces represent the achieved implant positions. Registration of the STL files of the surgical plan and postoperative scan (**A**), superimposition of the STL files of the implant and scan bodies (**B**, **C**), and data acquisition (**D**)

The primary outcome variables describing implant placement accuracy were angular deviation, coronal global deviation, and apical global deviation. The primary outcome variables were calculated by an investigator (A.T.) blinded to the modality used for implant placement during the model surgery. Planned implant positions relative to the surface of the models were exported using the Virtual Planning Export option of the coDiagnostiX 10.6 (Dental Wings, Montreal, Canada, USA) software in STL file format. Variables were calculated using Slicer 5.2.2 (The Brigham and Women’s Hospital, Inc., Boston, USA) software following the registration of the STL files of the surgical plan, postoperative scan, and implants and scan bodies [27]. Figure 2 shows the measurement of the outcome variables.

### Statistical analyses

Statistical analyses were performed using IBM SPSS software, version 28 (IBM Corporation, New York, NY, USA). According to the grouping by surgical modality used (pilot-guided with straight implant drills, pilot-guided with step drills, or fully guided approach), the Shapiro–Wilk test of the primary outcome variables was performed. The test revealed that all outcome variables were approximately normally distributed. One-way ANOVA with a post hoc test (Tukey) was carried out to compare angular deviation, coronal global deviation, and apical global deviation data among the three study groups. Values of p < 0.05 were considered statistically significant.

## Results

In each study group, dental implant placement was performed on five models, and six implants were placed per model, for a total of 90 implants placed in this study. Additional file 1 contains the primary data obtained from the trueness evaluation.

Pilot-guided implant placement followed up with step drills (Group 2) showed significantly lower values of both global deviation variables (coronal global deviation, 0.78 ± 0.29 mm; apical global deviation, 1.02 ± 0.56 mm) than pilot-guided implant placement followed up with straight drills (Group 1) (coronal global deviation, 0.95 ± 0.20 mm; apical global deviation, 1.42 ± 0.49 mm). However, there were no significant differences in angular deviation between Groups 1 and 2 (7.56 ± 2.92° and 6.44 ± 2.84°).

**Fig. 3:**
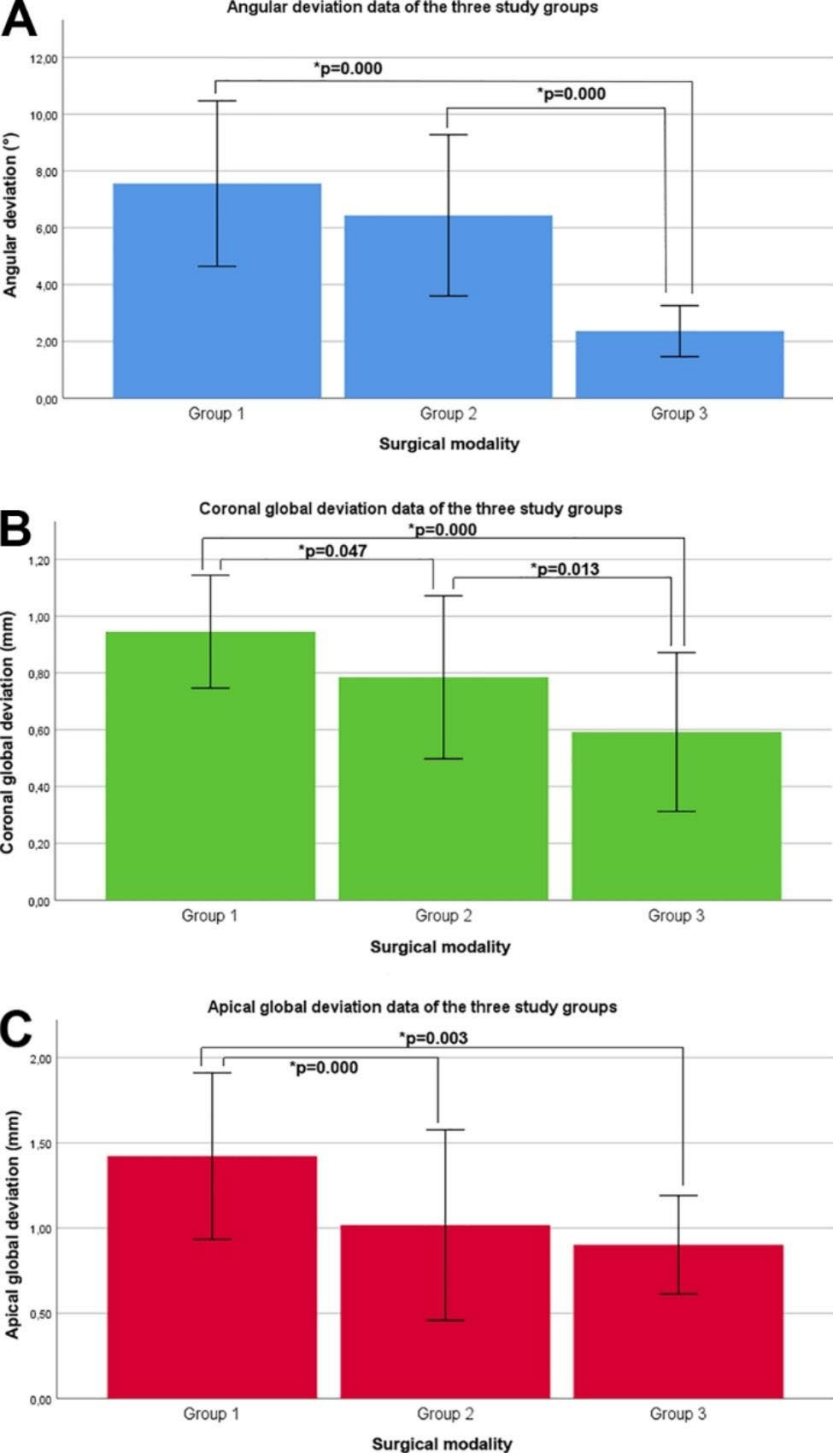
Bar graphs representing the angular deviation (**A**), coronal global deviation (**B**), and apical global deviation (**C**) data (mean ± standard deviation) with the results of the Tukey post hoc test. * p < 0.05. ± 1 SD is indicated by the error bars

The fully guided approach (Group 3) produced significantly lower values of all three primary outcome variables (angular deviation, 2.36 ± 0.90°; coronal global deviation, 0.59 ± 0.28 mm; and apical global deviation, 0.90 ± 0.29 mm) than Group 1 and significantly lower angular deviation and coronal global deviation values than Group 2. However, there was no significant difference in apical global deviation between Groups 2 and 3. Figure 3 presents the results of the statistical analysis.

## Discussion

Static CAIS is performed in clinical practice to enable dental implant placement close to the virtually planned, prosthetically desirable position [13, 14, 17]. Both fully guided and pilot-guided sCAIS significantly reduce the time of surgery and postoperative morbidity compared to free-hand implant placement [8, 11]. According to reviews conducted on the topic, further in vitro and clinical studies are needed to separately assess the number of factors in the digital workflow that may contribute to the deviation of achieved implant positions from the surgical plan [15, 28]. This is the first study to assess the influence of the implant drill design used to shape the implant bed during pilot-guided sCAIS on the accuracy of implant placement.

In the present study, improved implant placement accuracy was observed in models where step drills were used to complete implant bed preparation following the guided pilot osteotomy compared to when straight drills were used. However, different drill designs did not significantly influence the angulation of the implants placed. Therefore, the null hypothesis of the present study was rejected: the design of the drills used to finalize implant osteotomies following pilot-guided sCAIS influences dental implant placement accuracy.

Dental implant manufacturers provide surgical trays with drills deemed the most suitable for preparation of the implant bed. In a pilot-guided protocol, only the first osteotomy is guided by the surgical template [12, 29]. Therefore, implant placement accuracy is dependent on the extent to which the clinician can maintain the angulation and position of successive drills. Manufacturers provide straight drills or step drills in their surgical trays for cylindrical implants. The diameter of the apical portion of the step drills corresponds with the coronal diameter of the preceding drill in the drilling sequence, allowing the surgeon to better maintain the angulation and position of the osteotomy. According to the results of the present study, using step drills to finalize the implant bed during pilot-guided sCAIS improves implant placement accuracy.

In their study, Guentsch et al. reported on the implant placement accuracy achieved using pilot-guided sCAIS. According to their results, an angular deviation of 2.83 ± 0.79°, coronal global deviation of 0.54 ± 0.16 mm, and apical global deviation of 0.97 ± 0.25 mm were found using straight drills to finalize osteotomies [30]. Spille et al. used a similar approach and reported an angular deviation of 2.67 ± 1.58°, coronal global deviation of 1.009 ± 0.415 mm, and apical global deviation of 1.068 ± 0.384 mm [31]. In their study, Abduo et al. reported an angular deviation of 6.76 ± 2.49°, coronal global deviation of 0.40 ± 0.24 mm, and apical global deviation of 1.27 ± 0.50 mm achieved using a similar extent of guidance and finalizing the osteotomies with straight drills [32]. Stünkel et al. used a similar approach and found a mean angular deviation of 4.6°, coronal global deviation of 0.62 mm, and apical global deviation of 0.825 mm [25]. The outcome measures of Group 1 in the present study are within the range of the results of previous studies.

In their study, Kivovics et al. performed pilot-guided sCAIS using step drills to finalize implant placement and reported an angular deviation of 3.21 ± 1.52°, coronal global deviation of 1.31 ± 0.42 mm, and apical global deviation of 1.38 ± 0.41 mm [26]. The results of Group 2 in the present study show higher implant placement accuracy than that of Kivovics et al., which may be explained by the presence of teeth on the models used in the present study that may have restricted movement of the template during the interventions.

In their systematic review and meta-analysis, Bover-Ramos et al. reported on the implant positioning accuracy achieved in in vitro studies. According to their results, an angular deviation of 3.13 ± 0.23°, coronal global deviation of 1.00 ± 0.08 mm, and apical global deviation of 1.23 ± 0.10 mm may be expected using fully guided CAIS in in vitro studies [33]. The outcome measures of Group 3 in the present study are within the range of the results of this meta-analysis.

The material and technology used to manufacture surgical templates greatly influences the accuracy of sCAIS. It is paramount to analyse the biomechanical properties (compression and tensile modulus) of the materials used to produce surgical guides and assess their structural changes following compression [21, 22]. In the present study, we used the same technology and material for the preparation of surgical templates to eliminate bias related to the mechanical properties of 3D-printed materials.

According to the literature, fully guided sCAIS provides superior accuracy compared to half-guided and pilot-guided approaches [7, 29]. Therefore, this modality was introduced in the present study as a positive control for the pilot-guided groups. The fully guided approach performed better in all outcome variables compared to the pilot-guided protocol with straight drills and showed significantly lower angular deviation and coronal global deviation values compared to the pilot-guided protocol with step drills. However, there was no significant difference in apical global deviation between fully guided sCAIS and pilot-guided sCAIS completed with step drills. The results of the present study support that fully guided implant placement increases placement accuracy compared to the pilot-guided approach and shows that the angulation of the implant is the outcome most improved by guiding every step of implant bed preparation and implant placement.

According to the literature, implant placement accuracy positively correlates with higher bone density [34]. A resin model with a homogenous structure mimicking D3-type bone according to the Misch classification was used in the present study to eliminate the influence of bone density on implant placement accuracy.

Other factors that influence implant placement accuracy in sCAIS are sleeve height, length of the guided channel, and free drilling distance [35]. In the present study, these values were standardized over all three study groups to eliminate consequent bias.

The present study has several strengths; the same implant brand with uniform diameter and length was used in all study groups, the technique and material used to manufacture the surgical guides were the same in all study groups, and measures were taken to minimize participant bias. Fully guided implant placement showed better implant positioning accuracy than the pilot-guided approaches in the present study, which supports the evidence in the literature [17]. However, during a fully guided intervention, the surgical template remains in place for the whole procedure, inhibiting the cooling of the drills, which may be a disadvantage of this method [18, 19]. Pilot-guided surgery enables abundant cooling during implant bed preparation and requires less sophisticated instrumentation [7]. Heating of the surgical guides during the intervention and consequent dimensional changes may have affected implant placement accuracy in the present study [20]. In fully guided surgery, the surgical template remains in place for the whole duration of the intervention, whereas during pilot-guided surgery, the surgical template is removed following the pilot osteotomy. Therefore, the implant placement accuracy of the fully guided group may have been affected to a greater extent than that of the pilot-guided groups, which may be a limitation of this study. Another limitation of the present study was that it was carried out with only one implant system. Another limitation may have been that the level where the step was located on the drill was constant to prepare an implant bed suitable for the specific dental implant used in this study, which may have influenced implant positioning accuracy as well. The results of this in vitro study should be cautiously applied to the more elaborate clinical environment. Further clinical studies may be required to assess the influence of drill design on the accuracy of dental implant placement using a pilot-guided sCAIS procedure.

## Conclusions

According to the results of this in vitro study within its limitations (only one implant system, with a set of step drills with constant step height used, and uneven heat distortion of the templates among the study groups), the design of the drills used to finalize implant osteotomies following pilot-guided sCAIS influences dental implant placement accuracy.

### Electronic supplementary material

Below is the link to the electronic supplementary material.

Supplementary Material 1

## Data Availability

All data generated or analysed during this study are included in this published article and its supplementary information files.

## List of abbreviations

CAIS: Computer-assisted implant surgery
CBCT: Cone beam computed tomography
CI: Confidence interval
DICOM: Digital Imaging and Communications in Medicine
sCAIS: Static computer-assisted implant surgery
STL: Standard Tessellation Language
